# Perceptions of a drug prevention public service announcement campaign among street-involved youth in Vancouver, Canada: a qualitative study

**DOI:** 10.1186/s12954-017-0132-7

**Published:** 2017-01-13

**Authors:** Lianlian Ti, Danya Fast, William Small, Thomas Kerr

**Affiliations:** 1 British Columbia Centre for Excellence in HIV/AIDS, St. Paul’s Hospital, 608-1081 Burrard Street, Vancouver, BC V6Z 1Y6 Canada; 2 School of Communication, Simon Fraser University, K9671-8888 University Drive, Burnaby, BC V5A 1S6 Canada; 3 Faculty of Health Sciences, Simon Fraser University, 11300-8888 University Drive, Burnaby, BC V5A 1S6 Canada; 4 Department of Medicine, St. Paul’s Hospital, University of British Columbia, 608-1081 Burrard Street, Vancouver, BC V6Z 1Y6 Canada

**Keywords:** Public service announcement, Public health communication campaign, Illicit drug use, Street-involved youth, Social suffering

## Abstract

**Background:**

Due to the popularity of public service announcements (PSAs), as well as the broader health and social harms associated with illicit drug use, this study sought to investigate how drug prevention messages found in the Government of Canada’s *DrugsNot4Me* campaign were understood, experienced, and engaged with among a group of street-involved young people in Vancouver, Canada.

**Methods:**

Qualitative interviews were conducted with 25 individuals enrolled in the At-Risk Youth Study, and a thematic analysis was conducted.

**Results:**

Findings indicate that the campaign’s messages neither resonated with “at-risk youth”, nor provided information or resources for support. In some cases, the messaging exacerbated the social suffering experienced by these individuals.

**Conclusions:**

This study underscores the importance of rigorous evaluation of PSAs and the need to consider diverting funds allocated to drug prevention campaigns to social services that can meaningfully address the structural drivers of drug-related harms among vulnerable youth populations.

## Background

During the past half-century, public service announcements (PSAs) to promote healthy behaviors have become increasingly popular, including PSAs focused on preventing drug use among youth, which target both young people and their parents [1]. PSAs can be described as advertisements or commercials that aim to provide information or advice about a particular health or social issue, or promote activities that serve the wider community [2].

Across various settings, the literature on the effectiveness of drug prevention PSAs reveals mixed results [2, 3]. Some have argued for the need for more rigorous research into the circumstances under which PSAs may be effective, including more nuanced considerations of audience demographics such as age, gender, ethnicity, culture, and socioeconomic status [3–8]. However, others have maintained that PSAs are an ineffective method to communicate health information [2, 9–11].

Many PSAs focused on preventing drug use among youth use fear-based appeals in an effort to motivate behavioral change. Fear-based appeals can be defined as messages that aim to motivate behavioral change by frightening or threatening target audiences with negative outcomes if unhealthy behaviors are initiated or continued, and recommendations are not followed [6, 12]. To date, literature on the effectiveness of fear-based appeals in PSAs has also revealed mixed results [4, 5, 12]. Many have argued that the arousal of fear through PSAs can foster feelings of antagonism, alienation, or resentment among target audiences [4, 13–15]. Additionally, it has been suggested that by focusing on, and to a certain extent caricaturing, negative outcomes, fear-based appeals can encourage stigmatizing and oversimplified understandings of those who “fail” to follow the recommendations outlined in the PSA, such as people who use drugs [2, 13–15]. Indeed, PSAs can powerfully shape the way viewers understand and articulate fundamental questions about health and social issues like addiction and youth drug use [16].

The present study sought to understand how one group of street-involved young people in Vancouver, Canada understood, experienced, and engaged with the images, ideas, and information found in two video PSAs released as part of the Canadian Government’s *DrugsNot4Me* campaign. Given the continued popularity of fear-based appeals, we also sought to investigate the potential effect of the campaign in preventing the initiation of more severe and potentially harmful forms of drug use among young people experiencing social, economic, and institutional marginalization. Despite the campaign’s perceived target audience of youth who are at high risk of initiating drug use but have yet to, we chose to discuss the campaign with street-involved young people who had already begun engaging in heroin, crack cocaine, and crystal methamphetamine use. While we recognize that this campaign was not specifically aimed at our study population, it was important to elicit these young people’s perspectives on the campaign because they were at one time part of a particularly “high risk” segment of the broader Canadian youth population targeted by the campaign. On the one hand, these young people arguably stood to benefit the most from an effective drug prevention mass media campaign; on the other, this group of young people would also likely be the most negatively impacted by an *ineffective* drug prevention mass media campaign. Additionally, we were interested in investigating the wider impacts of the *DrugsNot4Me* campaign on individual and collective understandings of youth drug use, from the perspective of marginalized young drug users themselves. Whether or not they had initiated more severe and potentially harmful drug use prior to first exposure to the *DrugsNot4Me* campaign, we asked youth to reflect on how the PSAs could shape their understandings of themselves and their lives in the context of current or past experiences of addiction, as well as broader public understandings of the “problem” of youth drug use.

## Methods

### The *DrugsNot4Me* PSAs

In 2009, as part of its National Anti-Drug Strategy, the Government of Canada released *DrugsNot4Me*, a drug prevention PSA campaign that primarily targeted youth. From 2007 to 2012, a total of CDN $29.8 million was budgeted for the Government’s Prevention Action Plan, which included the *DrugsNot4Me* campaign [17]. The campaign centered around two video PSAs: *Fast Forward* [18] and *Mirror* [19], featuring a young male and female character, respectively. From 2009 to 2012, *Fast Forward* and *Mirror* appeared frequently on television and were made available on the campaign’s website (which also included information about illicit drugs and their effects directed at various audiences, including young people, parents, and educational institutions). The *DrugsNot4Me* campaign was also widely disseminated via print PSAs on public transit. The print PSAs featured the tagline, “Drugs, do you know where they’ll take you?,” embedded within stills of either the young male or female lead from the video PSAs.

The video PSA *Fast Forward* begins with a scene of a house party. Inside the house, there is loud music playing, and we see a group of teenagers dancing. The primary character, a young Caucasian male in his early teenage years, moves through the crowd. The next scene is of a small group of teenagers congregating outside of the house, smoking a “joint” of what we assume is marijuana. The primary character is offered the joint. The PSA then cuts to a rapid series of different scenes, in which the primary character is imagining the consequences of accepting the offer. These include a scene where he is taking what appears to be ecstasy (pills with “smiley faces” on them); a scene of him having a heated argument with his mother and scaring his younger sister; a scene of him sleeping during class while in school; a scene of him experiencing significant mental distress in his bedroom at home; and, finally, a scene of him getting caught with a bag of marijuana at school by an authority figure, presumably a teacher or principal. Following this, the PSA cuts back to the primary character refusing the offer of the joint. In the final scene, he is shown inside the party, laughing and talking with a different group of teenagers. A male voice narrates: “Drugs. Do you know where they’ll take you? To learn the effects of drugs and how you too can say no, visit *drugsnot4me* [dot] *ca*” [18].

The video PSA *Mirror* follows a young Caucasian female. The majority of the PSA takes place in her bedroom, and depicts her physical and psychological demise over time as a result of substance use. Her transformation is signaled by progressive changes in physical appearance (including her hairstyle, make-up, clothing, and personal hygiene), as well as her behavior, which becomes increasingly angry, erratic, and desperate. The PSA includes a musical rhyme, reminiscent of a children’s nursery rhyme, in which a female voice sings: “One, two, kicked out of school. Three, four, snort some more. Five, six, need my fix. Seven, eight, it feels too late.” At the end of the rhyme, we are shown a before-and-after image, in which the still healthy and attractive teenage girl sees her emaciated, dirty, and distressed “addict self” reflected in the mirror. Her reflected self has sores on her arms and mouth, implying the use of more severe drugs such as crystal methamphetamine and crack cocaine. The PSA closes with a scene of the young woman smiling and laughing as she leaves her house, accompanied by two other teenagers. A male voice completes the scene with the same narration as above (Government of Canada, 2009b).

### Study sample, data collection, and data analysis

Study participants were recruited from the At-Risk Youth Study (ARYS), an ongoing prospective cohort study of street-involved and drug-using youth in Vancouver, Canada [20]. Twenty-five in-depth, semi-structured, qualitative interviews were conducted with 11 women and 14 men. Interviews were conducted retrospectively, a number of years after the release of the *DrugsNot4Me* campaign, rather than as part of a prospective evaluation. Participants ranged in age from 20 to 32 years, with a median age of 25 years. Of the participants that self-identified as a single race or ethnicity, ten (40%) self-identified as White, five (20%) as Aboriginal, two (8%) as Southeast Asian, and one (4%) as Black/African-Canadian. Of the participants that self-identified as more than one race or ethnicity, five (20%) self-identified as Aboriginal and White, one (4%) as Aboriginal and Black/African-Canadian, and one (4%) as White and Black/African-Canadian. Each interview lasted approximately 1 h and was conducted by two female researchers (LT and DF) at the ARYS frontline office in downtown Vancouver between April and June of 2015. All participants provided written informed consent and received a thirty-dollar honorarium for their participation. The study was undertaken with ethical approval granted by the Providence Healthcare/University of British Columbia Research Ethics Board.

The study participants were not required to have seen the *DrugsNot4Me* video PSAs prior to the interview, and were shown both *Fast Forward* and *Mirror* several times throughout the interview process. However, the interviews revealed that the *DrugsNot4Me* campaign had a high level of exposure among the study participants. Of the 25 study participants, 23 (92%) indicated that they had been exposed to the campaign prior to the interview, while two (8%) indicated that they had no previous exposure. Participants indicated that they had been exposed to the campaign via multiple channels of communication (e.g., television, Internet) and in multiple locations (e.g., at home, on public transit, in youth shelters).

During interviews, we asked young people to describe their exposure to the campaign, and reflect on how it did or did not impact their own drug use-related decision-making across time. We asked them to tell us how the visual and audio components of the PSAs contributed to the specific stories being told and how the PSAs could impact individual and collective understandings of youth drug use in Canada. We also asked youth for their perspectives on the use of fear-based appeals in drug prevention messaging, and to compare the *DrugsNot4Me* video PSAs with video PSAs from other campaigns they had been exposed to. Finally, we asked young people for their recommendations on how to improve drug prevention PSAs, as well as their ideas about alternatives to drug prevention PSAs for addressing youth drug use.

All interviews were audio-recorded and transcribed verbatim, and transcripts were checked by the lead author to ensure accuracy. Following transcription, a coding framework was generated that captured broad, emergent themes (e.g., exposure to the *DrugsNot4Me* campaign*)*; this framework was then refined by the research team through the addition of new codes that captured more complex analytic categories (e.g., PSAs and social suffering). NVivo software was used to facilitate data coding and management.

## Results

### Unrealistic and decontextualized representations of drug use

Overall, findings of the present study indicated that the *DrugsNot4Me* campaign did not affect young people’s drug using behaviors, whether in terms of preventing the initiation of more severe forms of drug use or motivating young people to stop using drugs. The majority of the study participants characterized the storylines and primary characters featured in the *DrugsNot4Me* video PSAs as unrealistic, using terms such as “inauthentic”, “simplistic”, “illogical”, and “ridiculous”. One aspect of the PSAs that participants found particularly unrealistic was the message that drug use in general inevitably results in dire consequences:

What really irks me about both of these ads though, is **they’re just saying drugs in general**, you know? It’s a very, very, very vague term. And its just, **the context goes out the window** when they play this video. It’s like, you know what? Your message is not clear. (Participant #12, Male, White)

Notably, the participants of this study did not consider marijuana smoking to be a harmful form of drug use, nor did they consider it to be a “gateway drug” to more harmful forms of drug use such as ecstasy (a transition which is depicted in *Fast Forward*). It is important to note that during the time these PSAs were on the air, marijuana was essentially being decriminalized in Vancouver, revealing a discrepancy between the Government of Canada’s *DrugsNot4Me* campaign and the drug policy climate in Canadian metropolitan urban settings like Vancouver. Since the campaign, the presence of medicinal marijuana has increased dramatically in Vancouver, and is easily accessible to most adults. Yet, of the latest series of drug prevention PSAs released by the Government, one *continued* to focus on the potentially dire consequences of marijuana use among young Canadians [21]. The other emphasized the harms of prescription drug abuse [22].

Another main issue identified by a large number of participants was that the PSAs failed to acknowledge or address the broader contexts of the primary characters’ progressive drug use and addiction, including the social, structural, and environmental factors that powerfully shape young people’s vulnerability to illicit drug use and related harms in particular places. These factors include experiences of social, economic, and institutional marginalization in communities, neighborhoods, and schools; unstable housing and homelessness; and exposure to trauma and abuse, including sexual and physical violence [20, 23, 24]. In both video PSAs, the primary characters are shown in significant mental distress as their drug use and addiction progress. However, study participants consistently emphasized that mental distress—as a result of problems at home and at school for example—is an important context for *initiating* drug use:

They’re trying to shove like, **years of like, drama and abuse** into like, five seconds. If I watched and had no idea what was going on [i.e., had no prior knowledge of how addiction progresses] I would be very confused, probably. I would have no idea why she’s freaking out or anything. It wouldn’t make any sense at all. It’s all implied. (Participant #11, Male, White)

A number of young people drew attention to the ways in which the *DrugsNot4Me* campaign attempted to depict a very particular “reality” about youth drug use. Both of the central characters in *Mirror* and *Fast Forward* appear to be from middle-upper class families; they have nice clothing, well-furnished bedrooms, and what appear to be stable homes. Importantly, both characters are Caucasian. A number of young people commented that this depiction of youth drug use—and of vulnerability to drug use—was not representative of the vast majority of Canadian teenagers who struggle with addiction, including, notably, young people of Aboriginal descent [25–27]. As one young woman explained about *Mirror*:

Well, she looks like a yuppie. She’s well-dressed, her hair is clean, she’s somewhat pretty-looking, she probably has a good home. **My bedroom never looked like her bedroom,** I’ll tell you that. I don’t relate to her at all ‘cause my mom’s a drug addict. She’s been a drug addict since I was little. I’ve been around drugs all my life. This is—no, this is not realistic. Like, what happened? What’s her stressor? Like, why does she have to use drugs? She doesn’t look like the kind of person that needs to use drugs. (Participant #21, Female, Aboriginal–White)

### Reinforcing suffering, stigma, and public fear

While most participants pointed to the various ways in which the PSAs were unrealistic, several youth simultaneously found that they were able to relate to certain aspects of the PSAs’ storylines—in particular, the spiraling, negative consequences of severe addiction, as depicted in *Mirror*. Most participants believed that the “addict self” depicted at the end of *Mirror* is using crystal methamphetamine (meth), because she is shown with sores on her arms and mouth consistent with the “skin picking” behavior that can accompany intensive meth use. For those participants who had been or were currently addicted to meth, or who had a friend in that situation, watching *Mirror* could be emotionally painful, and reinforce feelings of sadness, alienation, and hopelessness. One participant expressed this through a retelling of her friend’s experience:

My friend really relates to that ad [*Mirror*] a lot. ‘Cause she’s from [a] well-off family. And like, that’s what happened to her. **She started crying** when she saw that commercial. It like, blew her out of the water. [It] gave her like, [a] kick in the ass. Yeah, she got really scared. I think [the ad is] sad. (Participant #6, Female, Aboriginal)

Similar sentiments were reflected in other participants’ accounts:

The [ads] just feel **painful**. I don’t like the sense of what’s going on. Sucks to watch [it], sucks to live it. (Participant #10, Male, White)

This is like, [a] really alienating ad, right here. It’s really making it seem like, there’s **no hope for that girl**, is what I’m getting. And that she’s fucked and you better not get to that point because there’ll be no hope for you as well. And there’s so may people that, in their addiction—whether it’s been a year or twenty years, they feel that way. And I feel like this is just backing that up. (Participant #17, Male, Aboriginal–White)

When asked to think about the impact of the PSAs more broadly, participants articulated a concern regarding the potential of the PSAs to create public fear surrounding youth drug use, and reinforce stigma and discrimination towards vulnerable young people who were not able to “just say no” to drugs. Regarding *Mirror*, participants noted:

[The ad] **makes society scared** of everybody that’s on drugs. You shouldn’t be afraid of it. I think you should just be more aware of it. More information on [the context of youth drug use] would be more helpful than to like, just put an ad on. Like, freak out people, you know? (Participant #6, Female, Aboriginal)

This ad is not solution-based in any way. It shows a problem, and [says], you know, prevent a problem by not doing it. But what if someone was to look at this ad and they are in that place in their lives? How is this going to be bettering [for] where they are in their addiction in any way? It’s just kind of creating a **separation**, making them appear to be in a different category. Like, the segregation of people who are on drugs. It’s just saying, ‘Don’t use drugs.’ It’s not saying that, you know, ‘But if you do use them, here’s how we can help.’” (Participant #17, Male, Aboriginal–White)

### Alternatives to fear-based drug prevention PSAs

When it comes to preventing youth drug use, almost all study participants argued that there are more effective alternatives to drug prevention PSAs that use fear in an attempt to influence or change individual behavior. With regard to developing more effective PSA campaigns, participants suggested that PSAs should be more informational and educational, providing facts about illicit drug use rather than emphasizing emotional storylines. It is important to note that the *DrugsNot4Me* campaign was not designed to address the needs of young people who were already engaged in more severe forms of drug use. Nevertheless, study participants consistently characterized the video PSAs as “problem-based” rather than “solution-based” and commented on how they promoted abstinence without offering useful information about how to access support for those young people struggling with addiction or the decision to use more severe and potentially harmful drugs. Though intended as a resource, the link to the *DrugsNot4Me* website featured at the end of the PSA was viewed as insufficient by participants. To this effect, one participant explained about *Mirror*:

[The ad] said *drugsnot4me.com*. If I got to learn more about that resource it would be more beneficial to me. Yeah, I would’ve liked to see more about what happens when I go to that site—what kind of help there is available to me. [Without it] I’m left with a story and a link. [It’s hard to] separate myself from the girl and imagine myself getting the help. [When she] goes onto the porch [of her home] and she’s with her friends—that’s great, but how did she get the help? And choosing not to use won’t just miraculously change your life. You can be sober and clean and still feel **dead on the inside** and still have all of the issues that you had when you were using. (Participant #14, Female, Aboriginal–White)

Study participants also suggested that the PSAs could be improved through the use of “real people with real stories”:

Maybe like, [if the ad] wasn’t fictional. That might help too—if it was like, actual people who’d gone through some of the things. ‘Cause that might resonate more with [people] than this fictional political ad created by the Government, you know what I mean? If it was like, ‘Oh, we interviewed some addicts and this is what they had to say’ and stuff like that. This is a real person—**we didn’t make this up to sell you an idea.** This is an actual person that’s gone through this. (Participant #3, Male, Southeast Asian)

One participant suggested this community-based, participatory approach to creating a drug prevention mass media campaign:

I think it would be more effective for them to hire onto their advertising team and their campaign team people who are recovered addicts. People who work in the addiction industry. People who have, like, **experience** being in and out of rehabilitation programs. And I feel like those people would have a lot of the similar ideas or thoughts that I have towards this and be able to really make an actual effective ad and program for people. (Participant #17, Male, Aboriginal–White)

However, the majority of participants were adamant that the taxpayer dollars invested in the production and dissemination of PSAs could have had more impact on youth drug use if it was used to provide different kinds of support to young people in crisis, such as detox facilities and harm reduction and mental health programs. Participants recognized the need for such programs to help those who are already addicted to drugs:

I would really want to focus on the drug addicts and not the could-be drug addicts. So what I would spend the money [used for the *DrugsNot4Me* campaign] on [is] **helping the already addicted.** More supportive housing, more mental health advocates. ‘Cause that’s what I see right now is the problem. The Downtown Eastside is [full of] drug addicts and, you know, drug addicts with mental health issues. ‘Cause I’m one of those people that have both. (Participant #23, Female, Aboriginal)

One participant in particular strongly emphasized the importance of providing community spaces and services targeted at vulnerable youth who have not yet initiated drug use, but are highly vulnerable to drug use. Briefly, he noted that while access to these kinds of spaces and services exists in downtown Vancouver, it is limited in other parts of the Lower Mainland and the Province of British Columbia. He described how a community center in his home town, which provided specific programing for youth (including leadership and volunteering opportunities), played a significant role in allowing him to move away from drug use and towards other opportunities:

I think the money [used for the *DrugsNot4Me* campaign] would be more useful going towards **services for youth** than the ads. I’m sure there are a lot of youth who don’t even pay attention to them or don’t fully understand them. I didn’t have access to things like a peer support worker or Coast Mental Health or anything like that [but] for me, it was the youth center [in the rural community where he grew up]. That was where I really started to take on like, leadership responsibilities and [started] volunteering and stuff like that. (Participant #9, Male, Aboriginal)

## Discussion

The Canadian government’s *DrugsNot4Me* drug prevention PSA campaign had an extensive reach. Indeed, this study provides evidence of its success in reaching some of Canada’s most vulnerable and marginalized young people. However, the results of the present study indicate that for this group of young people, the PSAs were simplistic and lacked information, and, for some, were even emotionally harmful. Importantly, many called for funding of drug prevention PSAs to be diverted to housing, mental health, and harm reduction programs. Our findings therefore support previous work which emphasizes the overall ineffectiveness of drug prevention PSAs, including those which use fear-based appeals [2, 9–11].

The findings of the present study are also consistent with previous work indicating that PSAs can have negative repercussions for vulnerable populations [2, 4, 13–15]. While the PSAs did not affect young people’s drug use practices, they did have a negative emotional impact on some participants and could actually exacerbate experiences of social suffering. Social suffering refers to how the collective *lived experiences* or *lived realities* of suffering can be powerfully shaped by social, structural, and environmental factors beyond individual control [28–30]. By espousing a simplistic, “just say no” approach to addressing the problem of youth drug use, the Government of Canada’s *DrugsNot4Me* campaign reduces drug use and its harms (e.g., physical and psychological demise) to personal choice. Reinforcing this personal choice narrative can deepen the stigmatization of those young people whose “choices” are powerfully constrained by broader social, structural, and environmental factors. This includes forms of self-stigmatization, through which young people blame themselves for not being able to “just say no” to drugs [31].

The potential negative impacts PSAs can have on vulnerable populations has led to arguments for the rigorous evaluation of PSAs and mass media campaigns in an effort to mitigate, or altogether avoid, these outcomes. However, despite the existence of policies around mandatory standardized post-campaign evaluation for media campaigns of $1 million or more in Canada, evaluative strategies often overlook the importance of pre-campaign evaluations, and neglect to measure the *emotional* impact campaign content can have on audiences, particularly when addressing issues related to vulnerable populations; rather, they focus on audience recall and understanding [32]. Further, the existing literature on PSAs reveals a lack of research into strategic methods by which these campaigns could be evaluated for both positive and negative impacts, including the emotional impact on targeted audiences and the potential to increase the stigmatization and self-stigmatization of vulnerable populations. The failure to develop and implement robust evaluations of public health communication campaigns during production and before dissemination represents an important area for future work [33–37].

Given the high costs associated with the production and dissemination of drug prevention campaigns, as well as the dire situation of many young people who use illicit drugs in Canada, the findings of the present study have implications for resource allocation. The *DrugsNot4Me* campaign was on the air from 2009 to 2012. From 2007 to 2012, the Government of Canada budgeted CDN $29.8 million for its Mass Media Campaign, as a part of its Prevention Action Plan [17]. From 2014 to 2015, the Government allocated CDN $5.5 million to Health Canada for the “prevention of illicit drug use” [38, 39]. These values indicate that the Government has placed significant emphasis on the production and dissemination of drug prevention mass media campaigns. However, our findings build on a growing body of evidence indicating that drug prevention mass media campaigns have very little impact on the drug using behaviors of vulnerable young people and may actually produce negative outcomes for youth who are already heavily stigmatized in society [2, 4, 9–11, 13, 14]. This includes not only the *DrugsNot4Me* campaign, but also the Government’s latest campaign, released in October 2015, which featured two PSAs about the harms of marijuana and prescription drug use targeted at parents [21, 22].

The need to consider the social, structural, and environmental contexts of health behaviors when designing a public health communication campaign is emphasized in various studies [3–6, 40–42]. It has been suggested that drug prevention PSAs should be conceptualized with a consideration of those factors external to the individual choice to “just say no” to drugs [2]. Ultimately, there is an urgent need change the ways in which PSAs are produced creatively if they are to continue to be employed. Funding for drug prevention PSAs should be contingent upon scientific evidence in support of their effectiveness and rigorous evaluation strategies; thus far, such evaluation and evidence has been lacking in Canada.

For many of our participants, the creation of drug prevention PSAs and mass media campaigns were not considered the most effective use of resources. Rather, participants suggested an alternative approach worthy of consideration: that funding for PSAs be reallocated to social housing, mental health, and harm reduction programs, as well as other community supports and services specifically designed for youth. Better supports and services for young people in the places of their childhoods are needed so that the “decision” to initiate more severe and potentially harmful forms of drug use does not come to seem like the only “decision” congruent with every day lived experience in the context of entrenched social, economic, and institutional marginalization [24, 43].

The present study has several limitations that warrant discussion. First, the thematic analysis conducted for the present study was focused on only one PSA campaign, and as such, the findings of this study may not apply to other drug prevention PSA campaigns or those on other health issues. Second, the findings revealed by this study are specific to the study participants and are not representative of the wider street-involved youth population in Canada or elsewhere. Third and relatedly, it is important to recognize that the campaign did not directly target our study population, street-involved youth, but rather youth who are potentially at high risk of initiation into drug use. As such, this study cannot speak to the impact of the campaign among its direct target audience. And finally, it is important to acknowledge the limitations of the interview process and the ways questions are framed, as well as the existence of power relations embedded in the research process, particularly when working with youth, that may influence responses to favor researchers’ interpretations [43].

## Conclusions

In conclusion, the images, ideas, and information found in the *DrugsNot4Me* campaign were understood, experienced, and engaged by participants in a way that reflects the lived experiences or realities [29, 30] of social suffering among young people who are street-involved and use drugs. Our findings ultimately support calls for interventions that address the social, structural, and environmental contexts that shape these young people’s lived experiences across the life course [44]. The obvious disconnect between the perspectives of the Government of Canada and street youth on what constitutes an effective drug prevention campaign necessitates consideration of alternative solutions to drug prevention PSAs and the use of fear-based appeals.

## Abbreviations

ARYS: At-Risk Youth Study
PSA: Public service announcement
